# Spatial structural characteristics of forests dominated by *Pinus tabulaeformis* Carr.

**DOI:** 10.1371/journal.pone.0194710

**Published:** 2018-04-13

**Authors:** Lianjin Zhang, Gangying Hui, Yanbo Hu, Zhonghua Zhao

**Affiliations:** 1 Experimental Center of Forestry in North China, Chinese Academy of Forestry, Beijing, China PR; 2 Research Institute of Forestry, Chinese Academy of Forestry, and the Key Laboratory of Tree Breeding and Cultivation, State Forestry Administration, Beijing, China PR; Tennessee State University, UNITED STATES

## Abstract

The Chinese pine (*Pinus tabulaeformis* Carr.) is an ecologically and economically important evergreen coniferous tree which dominates warm temperate forests throughout northern China. We established two permanent plots within the Chinese pine forest in the Jiulong Mountains, Beijing, China. To understand the structural characteristics and dynamics of these plots, we analyzed the spatial structural characteristics within nearest-neighbor relationships using the bivariate distributions of the stand spatial structural parameters: uniform angle index, *W*; mingling index, *M*; dominance index, *U*; and crowding index, *C*. Results revealed that most trees in the forest were randomly distributed. The predominant individuals and randomly arranged trees were in very dense areas and surrounded by the same species. In addition, both plots exhibited a uniform size differentiation pattern. The two plots differed mainly in the level of species mixture and dominance. The majority of reference trees in the pure Chinese pine forest (plot 1) exhibited poor species mingling and low dominance, whereas trees in the mixed Chinese pine forest (plot 2) were evenly distributed in each mingling class and most trees were of intermediate dominance. The study results are useful for optimizing forest management activities in the studied stands, promoting tree growth, regeneration and habitat diversity, and improving forest quality at a fine scale.

## Introduction

Forests are three-dimensional systems whose biophysical structure plays an important role in ecosystem functioning and diversity [1,2]. Forest structure is both a product and driver of ecosystem processes and biological diversity [2]. It reflects both autogenic developmental processes, such as the regeneration pattern, competition, and the consequent self-thinning, and past and present disturbance events. Thus, forest structure has become an important factor when analyzing and managing forest ecosystems.

Forest spatial structure describes the spatial relationships among different species in the same forest community. In other words, it is the spatial distribution of tree positions and their attributes. Forest spatial structure provides a more detailed description of a forest and largely determines the properties of the system as a whole, including total biomass production, biodiversity, habitat functions, and the quality of ecosystem services [3]. It is a significant component of forest structure and has been used to monitor the spatial and environmental heterogeneity, identify the niche requirements of tree species, assess the temporal and spatial dynamics of vegetation, identify the effects of inter- and intra-specific competition, predict forest productivity, explain climate-related changes, provide valuable information for more adaptive and sustainable forest management, and investigate hydrological processes [4,5]. The accurate and effective description of spatial structure has attracted the attention of scholars [6].

Various qualitative and quantitative indices have been developed to describe and compare stand spatial structures, such as the index of Clark and Evans [7], the diffusion index [8], Ripley’s K-function [9], the K-function [10], the O-ring statistic [11], the Gini coefficient [12], Pielou’s isolation index [13], the mean directional index [14], Gadow’s species mingling [15], the mixed ratio [16], and the uniform angle index [6,17]. Some of these indices have been used widely in forestry and ecology. Many of these indices are related to the spatial relationships between neighboring trees.

In recent years, a set of structural parameters that reflects the nearest neighbor relationships between a reference tree and its four nearest neighbor trees has attracted attention for analyzing the characteristics of spatial structure and competition, calculating dominance and species diversity, adjusting structure, and guiding good forestry practices [16,18–28]. This approach uses four indices: uniform angle (W), mingling (M), dominance (U), and crowding (C) indices. The W index reflects the degree of distribution regularity. M is the similarity probability of tree species. U indicates the relationships of tree size. C is the degree of crowding of the neighbors surrounding the reference tree. Compared with traditional methods, this method has many advantages that involve using frequencies to express the attributes among trees [29–32].

The Chinese pine (*Pinus tabulaeformis* Carr.) is an endemic dominant species of temperate warm forests in China that grows mainly in northern China [33]. It grows on more than 2.5 million ha, with an estimated stocking volume of 0.13 billion m^3^ [34]. Chinese pine occupies a very important position in mountain vegetation restoration and has invaluable ecological, social, and economic benefits. Due to insufficient planting and the need for extensive management with a long time lag, Chinese pine plantations have done poorly and have low ecological function. Therefore, the scientific management of Chinese pine plantations has become a top priority. A comprehensive analysis of stand structure is an important basis for scientific management. Although many structural characteristics of this species (age structure, distribution pattern, species diversity, etc.) have been published [35–38], they mainly use the non-spatial structure or one-dimensional distribution of spatial structure to analyze the overall characteristics.

In comparison, this study examined the spatial structure characteristics of Chinese pine forests based on the relationships with neighboring trees. To clarify the ecological characteristics, the stability and succession of Chinese pine communities, and the formation and maintenance of Chinese pine ecosystems, we analyzed the spatial structure characteristics of two types of forest stand dominated by Chinese pine. This study explored the community structure characteristics of pure and mixed Chinese pine forests and the structural differences between pure and mixed Chinese pine forests. It also discusses possible methods for increasing the stability of Chinese pine plantations.

## Materials and methods

### Ethics statement

This research was conducted in Experimental Center of Forestry in North China, Chinese Academy of Forestry (ECFNC for short). This study was also supported by this center. We confirmed that the location is not privately owned and the trees investigation was approved by ECFNC. We also confirmed that the field studies did not involve endangered or protected species.

### Study area

**Fig 1:**
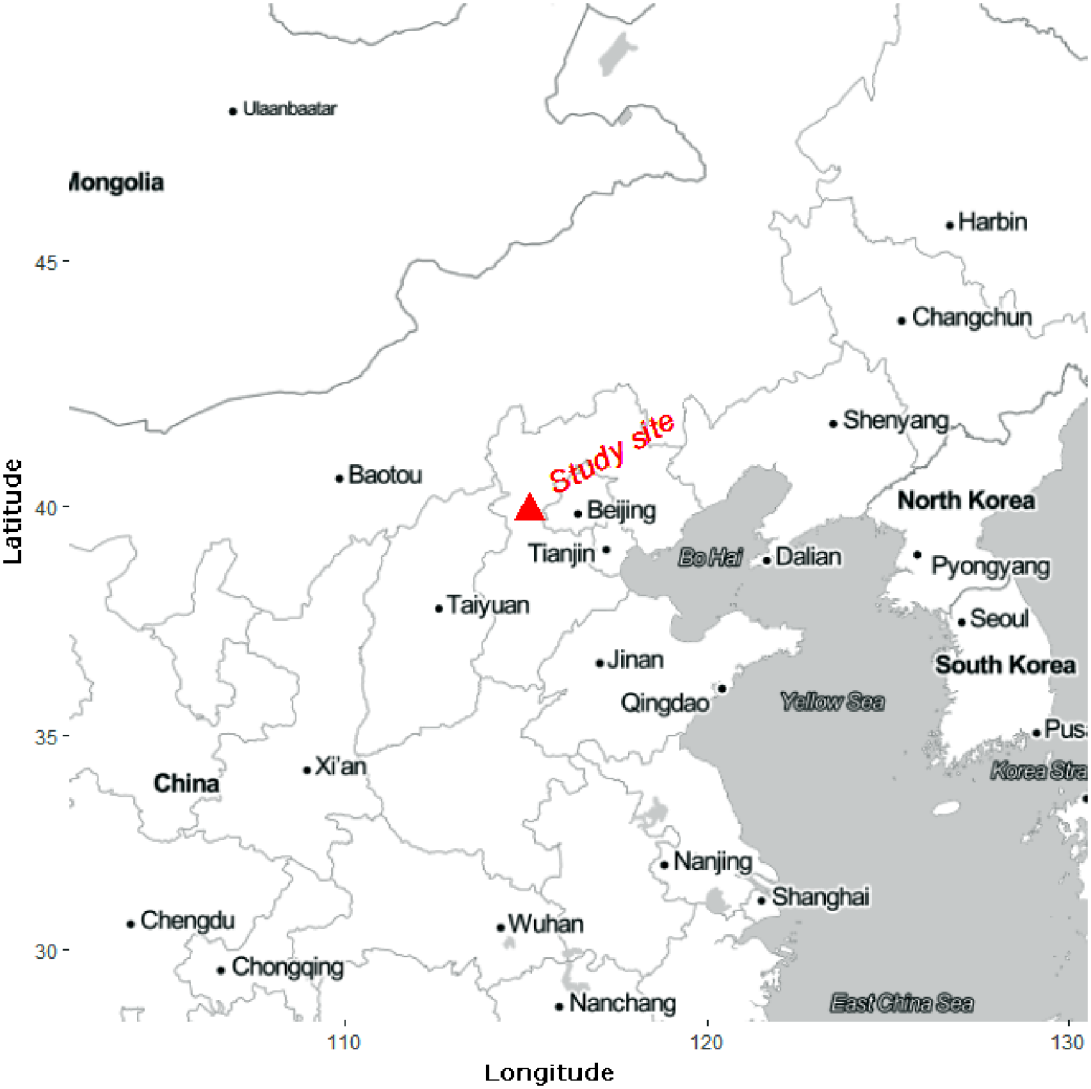
Map of the study site. The map was produced using the Ggmap package in R [40].

This investigation is based on observations made in forest stands dominated by *P*. *tabulaeformis* in the Jiulong Mountains (ll5°59′–ll6°07′E, 39°54′–39°59′N), which are located near Beijing east of the Taihang Mountains (Fig 1). The climate in this region has been classified as temperate continental affected by monsoon climate. The elevation in the area ranges from 100 to 997 m. The annual average temperature is 11.8°C. The region receives an annual mean precipitation of 630 mm and has an average yearly relative humidity of 66%. The distribution of the precipitation is relatively uneven during the year, with a relatively wet season from June to September and a relatively dry season beginning in October and ending in May of the following year. The total evaporation capacity and frost-free period are approximately 1870 mm and 216 days, respectively. The soil is a brown rocky mountain forest soil with a high stone content and the average soil layer thickness is 20 to 50 cm. Furthermore, the topography is steep and undulating [39].

### Field sampling

**Fig 2:**
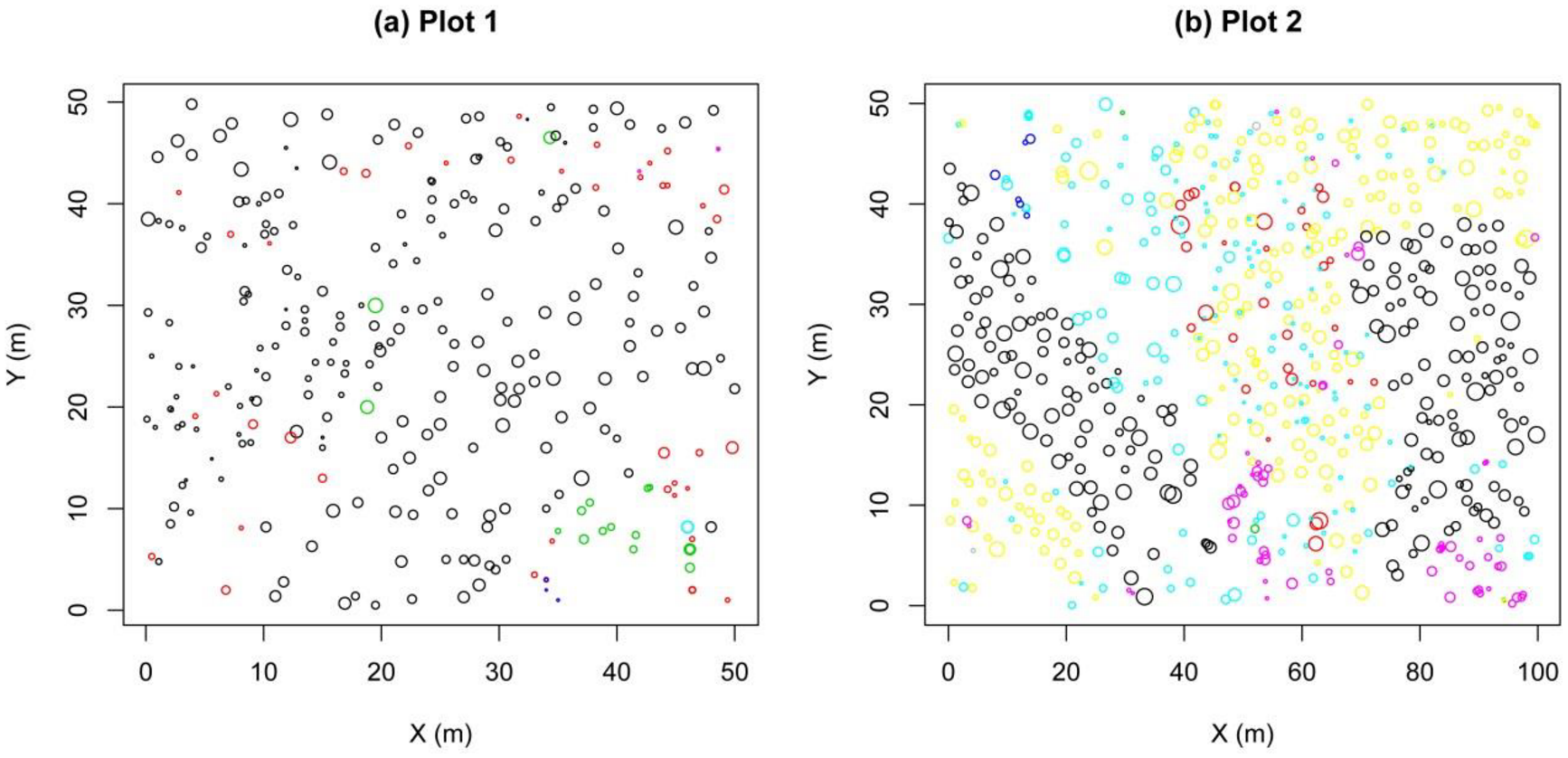
Observed tree point patterns in plots (a) 1 and (b) 2. Tree species are indicated by circles of different colors. Symbol sizes are proportional to the diameter at breast height (DBH). X and Y are the perpendicular coordinate axes of the plots.

Two permanent plots were established in the summer of 2015. The pure forest site covered 2500 (50 × 50) m^2^ and the mixed forest site 5000 (50 × 100) m^2^. Both plots were dominated by *P*. *tabulaeformis*. The main canopy species in the pure forest (Plot 1) were *P*. *tabulaeformis* and *Ulmus pumila*, while those in the mixed forest (Plot 2) were *P*. *tabulaeformis*, *Larix principis-rupprechtii*, *Syringa pekinensis*, and *Tilia mandshurica*. In each plot, all of the trees with a diameter at breast height (DBH) >5 cm were tagged, and their positions were mapped with a Topcon GTS602 (Topcon, Tokyo, Japan) autofocus total station. The tree DBH, height, and crown diameter were recorded S Data. Fig 2 shows the spatial distributions of the trees in both plots [40]. Table 1 provides general information on the plots.

**Table 1:** Stand characteristics of the two plots.

Plot	Slope (°)	Mean Altitude (m)	Canopy Cover	Tree Density (trees/ha)	Mean Basal Area (m^2^/ha)	Mean DBH (cm)	Number of Species
Plot 1	25	685	0.80	1148	17.8	14.0	6
Plot 2	23	725	0.85	1350	25.5	15.5	11

### Data analysis

#### Stand structural parameters based on neighborhood relationships

**Fig 3:**
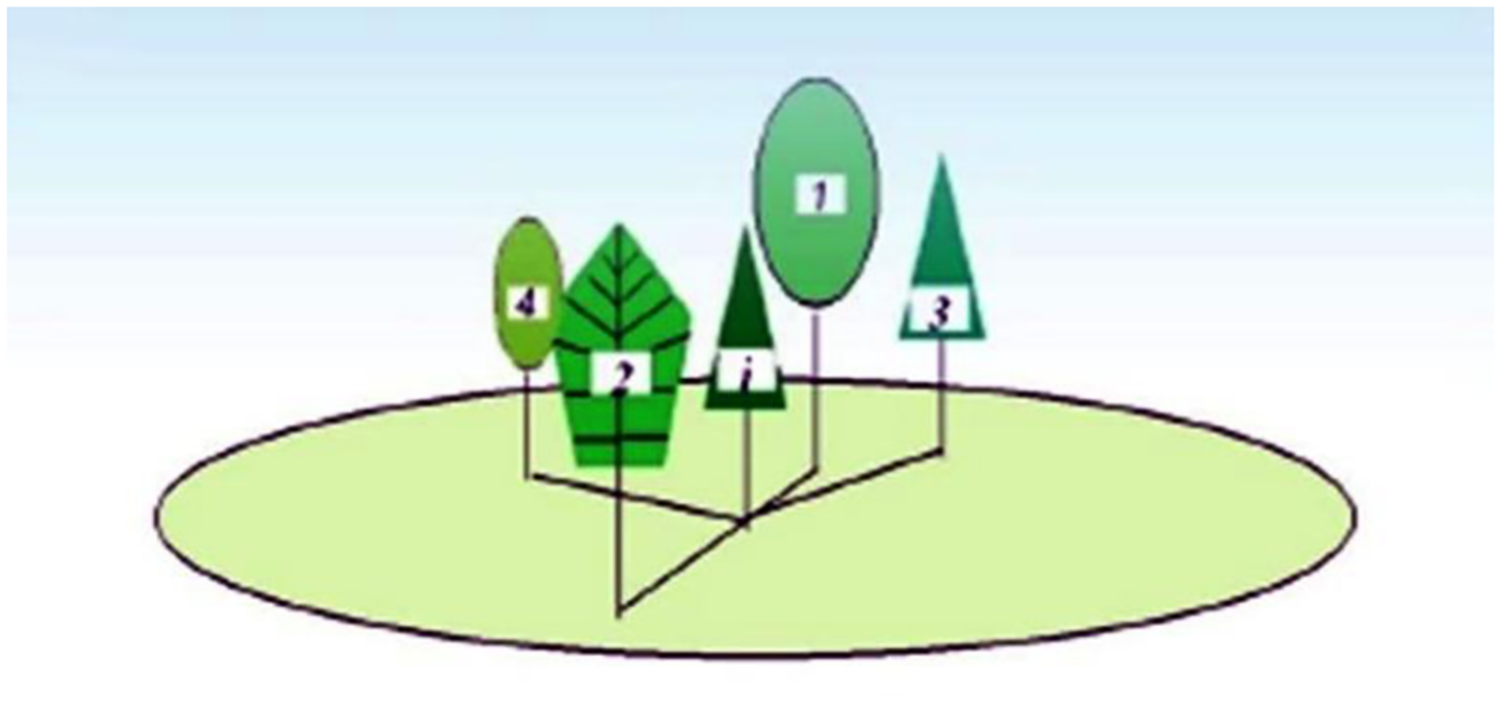
The structural unit.

**Fig 4:**
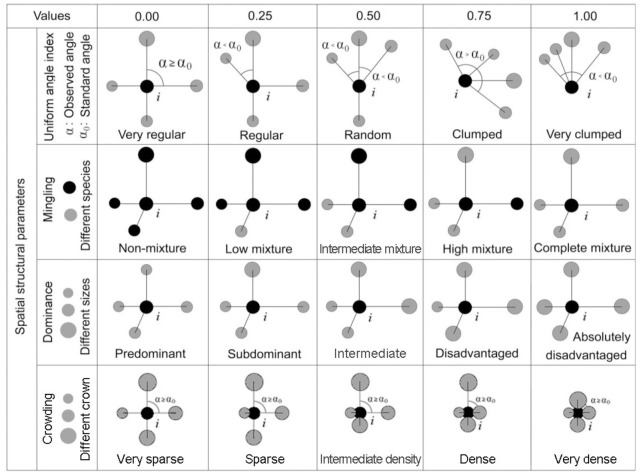
Specific characteristics of the four stand structural parameters.

The ‘structural unit’ was defined as a neighborhood involving a focal tree and its four nearest neighbors (Fig 3). Any structural unit can be synchronously described by multiple factors, such as tree size (DBH or crown), tree species, and tree distribution in the space. These factors can be readily expressed by a group of stand structural parameters (Fig 4): the W index [3,32], M index [16,31], U index [41,42], and C index [27]. These four parameters have obvious biological significance and can be easily and rapidly obtained in the field.

The W index, which is defined as the proportion of the angles *α* that are smaller than the standard angle *α*_0_(72°), is calculated as:

$$W_{i}=\frac{1}{4}\sum_{j=1}^{4}z_{ij}$$ (1)

Where *z*_*ij*_ = 1 if *a* < *a*_0_, and *z*_*ij*_ = 0 otherwise.

The W index indicates the spatial dispersion of the four nearest neighbors around the reference tree. Increasing values indicate a transition from regular to random to clumped spatial pattern.

The M index reflects the probability that the reference tree belongs to the same species as its four nearest neighbors and can be calculated as:

$$M_{i}=\frac{1}{4}\sum_{j=1}^{4}v_{ij}$$ (2)

Where *v*_*ij*_ = 1 if the *j*^th^ neighboring tree is not of the same species as the *i*^*th*^ reference tree, and *v*_*ij*_ = 0 otherwise. A higher value implies more species in the structural unit.

The U index reflects the relationship between the size of the reference tree and its four nearest neighbors and is defined as:

$$U_{i}=\frac{1}{4}\sum_{j=1}^{4}k_{ij}$$ (3)

Where *k*_*ij*_ = 1 if the *j*^th^ neighboring tree is smaller than the *i*^*th*^ reference tree, and *k*_*ij*_ = 0 otherwise. A higher value implies that the reference is larger (dominant) than all four neighbors.

The C index reflects the relationship between the canopy of the reference tree and its four nearest neighbors and can be expressed as follows:

$$C_{i}=\frac{1}{4}\sum_{j=1}^{4}y_{ij}$$ (4)

Where *y*_*ij*_ = 1 if the canopy projection of the *j*^th^ neighboring tree overlaps that of the *i*^*th*^ reference tree, and *y*_*ij*_ = 0 otherwise. The C index reflects not only the degree of crowding of trees and their four nearest neighbors with competition information, but also whether the forest canopy layer covers the woodland continuously. The greater the cumulative value of C, the higher the stand density, and the more continuous the coverage of the canopy is.

#### The bivariate distribution characteristics of the stand structural parameters

Bivariate distributions were studied involving the following six pairs of stand structural parameters: mingling-dominance (M-U), mingling-uniform angle index (M-W), mingling-crowding (M-C), dominance-uniform angle index (U-W), dominance- crowding (U-C), and uniform angle index-crowding (W-C). Each joint probability of the bivariate distribution contains 25 structural combinations.

## Results

### *M*-*U* bivariate distribution

**Fig 5:**
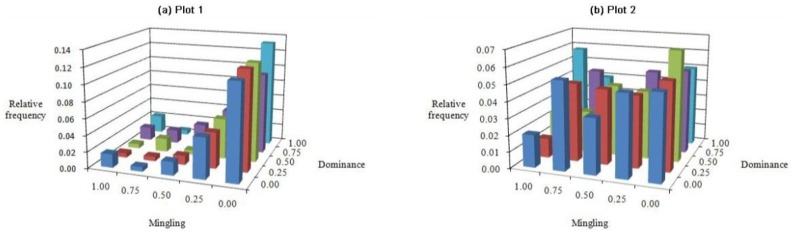
Bivariate distributions for mingling *vs*. dominance: Plots (a) 1 and (b) 2.

One feature of the M-U bivariate distribution (Fig 5) common to each plot was that the frequency values for each class of U (U = 0.00–1.00) were approximately the same. By contrast, the frequency values for each grade of M (M = 0.00–1.00) differed completely. Of the trees in plot 1, a proportion of 0.824 showed low M (M = 0.00–0.25) (Fig 5a), whereas the trees in plot 2 were evenly distributed at each grade of M (Fig 5b). Specifically, most reference trees in plot 1 are surrounded by the same species. Another different feature of each plot was that the frequencies of trees in plot 1 increased with decreasing M levels, with a maximum proportion of 0.132 at structural combination (M = 0.00, U = 1.00). The maximum value was three times greater than that of the other combinations, which had a mean frequency < 0.037 (Fig 5a). By contrast, with the exception of the structural combination (M = 1.00, U = 0.75), the frequency values of trees in plot 2 changed between 0.10 and 0.60, and no trend was obvious. It peaked (0.068) at the combination (M = 0.00, U = 0.50) (Fig 5b).

### *M*-*W* bivariate distribution

**Fig 6:**
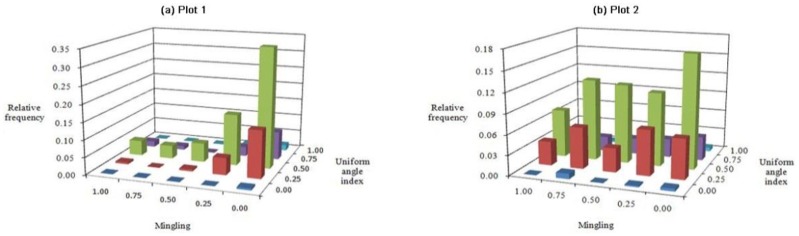
Bivariate distributions for mingling *vs*. uniform angle index: Plots (a) 1 and (b) 2.

The M-W index models (Fig 6) show that the highest frequency in the two plots was where the M index = 0.00 and the W index = 0.50. The frequencies were 0.346 and 0.169 for plots 1 and 2, respectively. In other words, the most common structural units were those in which the tree species of all four neighbors were the same as that of the reference tree and had a random distribution pattern in the quadrats. Another common feature of each plot was that more than half of the trees were randomly distributed (W = 0.50) and the number of individuals with a regular distribution (W = 0.00–0.25) exceeded the number of individuals in clumps (W = 0.75–1.00). However, plots 1 and 2 differed in that approximately 1.2 and 2 times as many individuals, respectively, had regular distributions than were in clumps (Fig 6a and 6b). The frequency values for each M class increased initially and then declined, accompanied by an increase in the degree of the uniform angle index.

### *M*-*C* bivariate distribution

**Fig 7:**
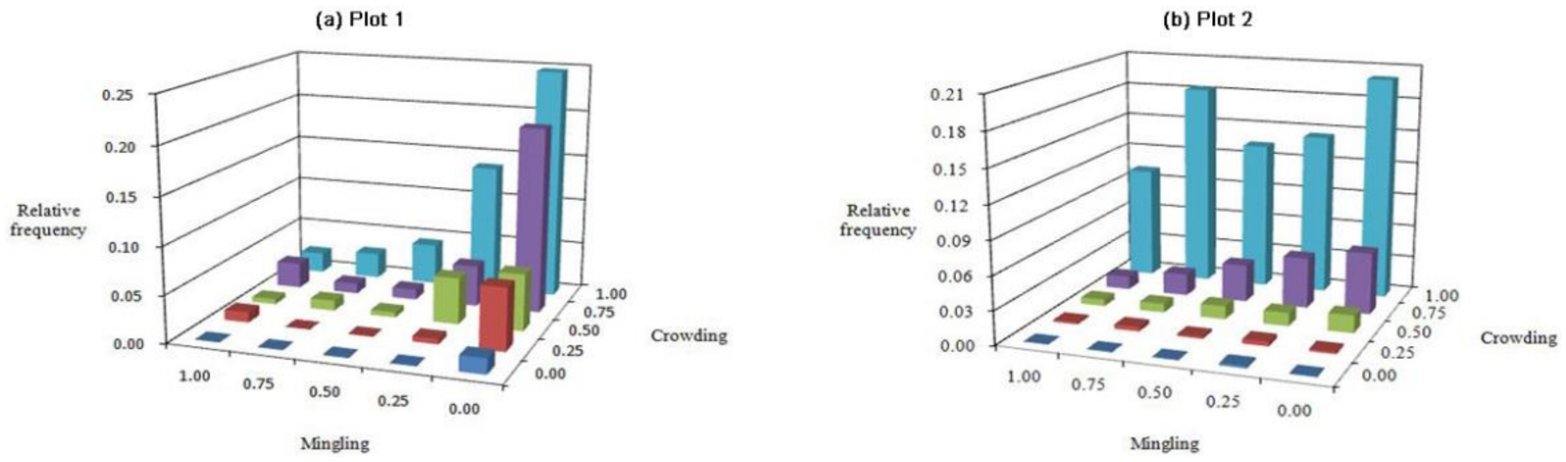
Bivariate distributions for mingling *vs*. crowding: Plots (a) 1 and (b) 2.

In the M-C bivariate distribution (Fig 7), the highest pole value of both plots always occurred with the combination of M = 0.00 and C = 1.00, which represents the case in which proximity of trees around the reference tree is very dense and it is surrounded by the same species. This case always had a frequency value > 0.200, and the mean value was six times greater than that of the other combinations, which had a mean frequency value less than 0.033. The frequency of each C class increased gradually from 0.00 to 1.00, and most of the individuals were parts of aggregations with high to very high degrees of C (C = 0.75–1.00); the frequencies were 0.769 and 0.930 for plots 1 and 2, respectively (Fig 7a and 7b).

### *U*-*W* bivariate distribution

**Fig 8:**
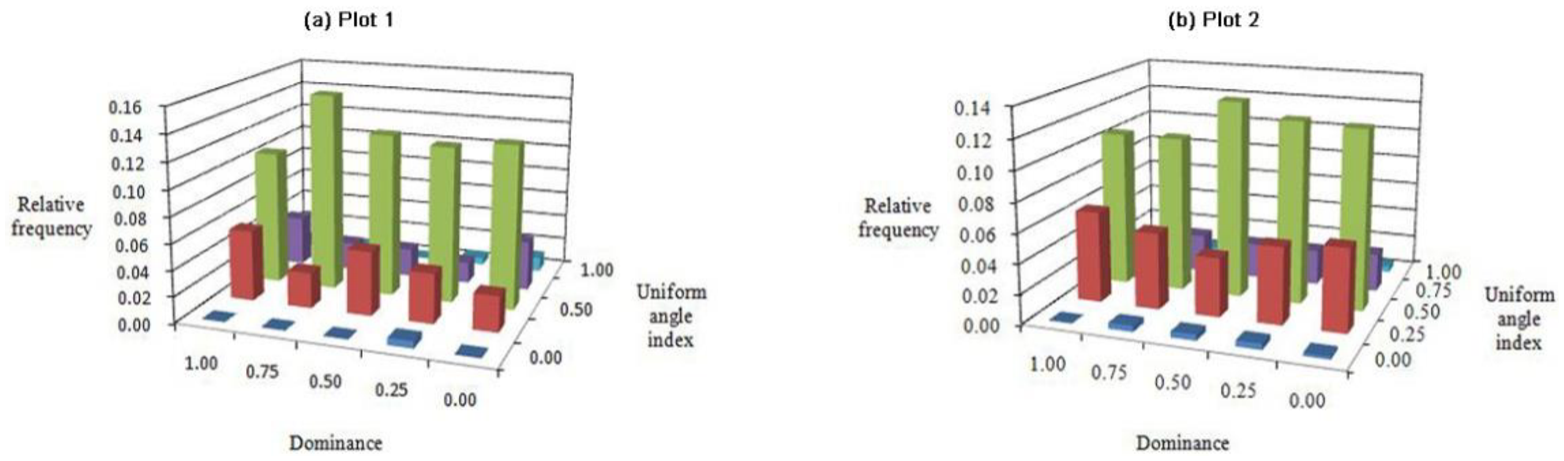
Bivariate distributions for dominance *vs*. uniform angle index: Plots (a) 1 and (b) 2.

The two plots had similar U-W index bivariate distributions (Fig 8). Namely, the U-W bivariate distribution was approximately symmetrical around the random distribution axis (W = 0.50) and declined gradually toward zero on both sides. The frequency of U increased with the W index and then decreased, suggesting a normal distribution. In addition, more than half of the frequency values fell along the random distribution axis (W = 0.50), and their frequencies in each row were between 0.104 and 0.154. When compared with the combinations in each plot with frequency values between 0.000 and 0.062, the high frequency of W index = 0.50 is notable, and accounted for 0.632 and 0.588 of the whole, respectively. One slight difference between the two plots was that the frequency value of plot 1 was 6% lower than that of plot 2 on the regular distribution axis (W = 0.25). The highest pole value appeared with different structural combinations: the highest frequency value of plot 1 was concentrated at the structural combination (U = 0.75, W = 0.50) (Fig 8a), whereas that of plot 2 was located at structural combination (U = 0.50, W = 0.50) (Fig 8b). In other words, most trees had random distributions; however, the former were in the disadvantageous U range, whereas the latter were in the intermediate U range.

### *U*-*C* bivariate distribution

**Fig 9:**
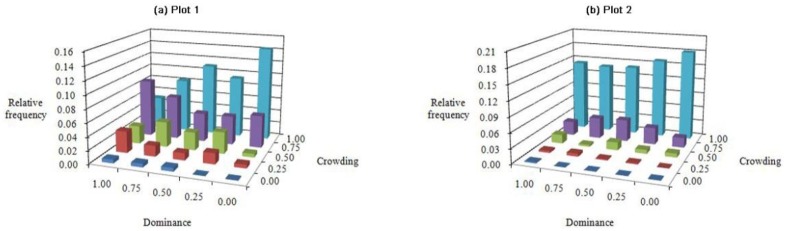
Bivariate distributions for dominance *vs*. crowding: Plots (a) 1 and (b) 2.

One common feature of the U-C bivariate distribution (Fig 9) in the two plots was that the highest frequency was at U = 0.00 and C = 1.00. The frequencies were 0.143 and 0.181 for plots 1 and 2, respectively. Namely, the most common structural units were those in which the reference tree was predominant and belonged to a very dense group. Another feature common to each plot was that the frequency values of U increased with the C level, and most individuals fell along intermediate-high degrees of C (C = 0.75–1.00). However, compared with plot 1 (Fig 9a), a majority of the frequency values (0.763) for plot 2 were aggregated at a high degree of C (C = 1.00) (Fig 9b).

### *W*-*C* bivariate distribution

**Fig 10:**
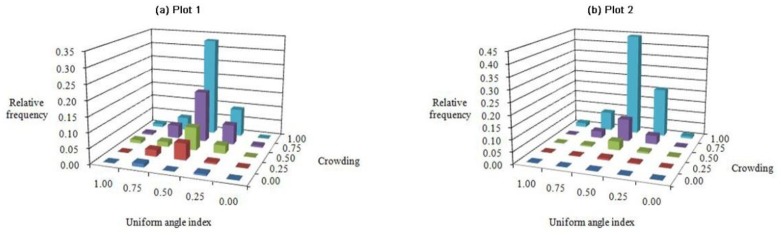
Bivariate distributions for uniform angle index *vs*. crowding: Plots (a) 1 and (b) 2.

The W-C bivariate distributions in both forests were similar (Fig 10). Most of the frequency values were located at the structural combination (W = 0.50, C = 1.00), accounting for 0.330 and 0.443 of the entire combination, respectively. That is, the most common structural units were those in which the reference tree was in a very dense group and had a random distribution pattern in the quadrats. The frequency values of the W index increased with the C level, and most individuals fell among intermediate -high degrees of C (C = 0.75–1.00). In addition, more than half of the frequency values fell along the random distribution axis (W = 0.50), and the closer the W index was to 0.50, the greater the disproportion.

## Discussion and conclusions

The bivariate distributions of forest stands dominated by Chinese pine revealed that the majority of reference trees were in very dense groups and randomly distributed, and that the tree size differentiation was not significant. It also demonstrated that the proportions of frequency values were distributed almost uniformly in each grade with a mean U value of nearly 0.20. The frequency of each C class increased gradually from 0.00 to 1.00. In addition, most trees were not mixed in the pure Chinese pine forest, and most neighbor groups consisted of five conspecific trees; whereas the trees were evenly distributed in each grade of M in the mixed Chinese pine forest (Figs 5–7). In a microenvironment, the frequency of U increased with the W index and then decreased, suggesting a normal distribution. The largest proportion of trees in the pure Chinese pine forest were at a disadvantageous dominance level and randomly distributed, whereas the largest proportion of trees in the mixed Chinese pine forest was in the intermediate U level and randomly distributed (Fig 8). Most of the predominant individuals and randomly arranged trees were very densely grouped and surrounded by the same species (Figs 7, 9 and 10). In other words, the difference in the six bivariate distributions of the stand structural parameters (M, W, U, and C) between the pure and mixed Chinese pine forests was mainly caused by the distribution of M. The pure and mixed Chinese pine forests were low- and intermediate-mixture states, respectively. These attributes indicate that Chinese pine forests have relatively low heterogeneity. The results provide valuable, detailed information on the structure of Chinese pine forests, which will help when developing sustainable management plans.

Chinese pine is famous for its ecological characteristics, including strong root systems and cold and drought resistance. Chinese pine forests have important ecological functions and include many warm-temperate components. Regionally, mixed forest containing *Quercus* is the top vegetation and climax community. Compared with natural forests of Chinese pine, the studied forests had lower spatial heterogeneity due to their artificial origin, disturbance history, and subsequent development. This difference was also related to their regeneration patterns and reproduction strategies. After the canopy closes, the competition among conspecific plants for the same resources gradually intensifies because of their similar requirements. Consequently, the weaker conspecific trees tend to die, resulting in self-thinning. These processes not only provide the necessary conditions for the invasion and growth of other species, but are also the primary reason why pure Chinese pine forest has some associated species. In addition, these results describe an ecological pattern in which the trees in Chinese pine forests are arranged randomly. Other researchers have found that the tree distribution pattern in Chinese pine forests changes constantly among different growth and development stages [35,43].

In this study, structural parameters were considered to be tightly associated with mixture, size differentiation, distribution patterns, and crowding between each individual and the four adjacent neighboring trees. These structural parameters have strong operability [3, 22–23, 41], which makes the precise adjustment of spatial structure possible. The bivariate distributions of the structural parameters are not only applicable to the spatial structure analysis of the community, but also to the structure analysis of tree populations [24]. This method should further our understanding of the diversity of population structure. Moreover, it is not necessary to conduct a comprehensive survey to obtain spatial information on specific species because this can also be achieved with a sample survey based on the tree structure unit [3,41], which can save much time and effort. Finally, analysis of the dynamic succession of a population may be part of future work by combining the bivariate distributions of structural parameters with different environmental factors.

The bivariate distributions of the structural parameters are useful for adjusting spatial structure and the optimization and restructuring of management practices. For example, they can be used to analyze selective harvest events in a continuous cover forest (CCF) management system. This method can provide critical and detailed information about spatial species mingling, distribution patterns, dominance, and crowding. Using the bivariate distribution characteristics, foresters can not only accurately determine the harvesting priority for trees, but also control the intensity of harvest. For example, according to the frequency distribution shown in Fig 6, trees with a low mixed and clumped status would be harvested to promote forest development towards a higher species mixture and random distribution. When it is necessary to promote forest development towards a higher dominance and less crowding, trees with low dominance and very dense status would be harvested according to the frequency distribution shown in Fig 9. Previous studies have confirmed the advantages of using the bivariate distributions of structural parameters. These advantages have been illustrated for harvesting [44, 45]. Daume used the bivariate distribution of mingling and diameter differentiation for selecting trees to harvest [44], while Li used the bivariate distributions of mingling, uniform angle index, and dominance for selecting trees to harvest [45].

The bivariate distributions of the structural parameters also have other advantages when modeling forest, *i*.*e*., they are affected less by forest type and status, more flexible for analyzing complex forests, more practical for forest management, and less expensive.

## Supporting information

S1 Data (XLSX)
